# Investigation into haemodynamic stability during intermittent haemodialysis in the critically ill

**DOI:** 10.1186/cc10978

**Published:** 2012-03-20

**Authors:** R Docking, L Moss, M Sim, D Sleeman, J Kinsella

**Affiliations:** 1 University of Glasgow, UK; 2 University of Aberdeen, UK

## Introduction

Studies that have reported cardiovascular (CVS) instability with haemodialysis (HD) are outdated and small. By analysing sessions in detail it will be possible to identify the frequency and nature of CVS instability. Hypothesis 1: haemodialysis is associated with CVS instability in the majority of sessions. Hypothesis 2: the majority of CVS changes in unstable sessions will be harmful/potentially harmful.

## Methods

Data were collected for 209 patients, identifying 1,605 dialysis sessions. Analysis was performed on hourly records, classifying sessions as stable/unstable by a cut-off >±20% change in baseline physiology (HR/MAP). Data from 3 hours prior to and 4 hours after dialysis were included, and average and minimum values derived. Three time comparisons were made: pre-HD:during, during HD:post, pre-HD:post-HD. If a session was identified as being unstable, then the nature of instability was examined by recording whether changes crossed defined physiological ranges. The changes seen in unstable sessions could be described as to their effects: being harmful/potentially harmful, or beneficial/potentially beneficial.

## Results

Discarding incomplete data, 1,563 sessions were analysed. A session was deemed to be stable if there was no change >±20% in time-averaged or minimum MAP/HR across three time comparisons. In 1,563 sessions there was stability in 874 sessions (55.8%, 95% CI for SEM 53.2 to 58.4). Hypothesis 1 is rejected. Each session had 12 potential comparisons of MAP, HR and time, therefore in the 689 unstable sessions there were 8,268 potential changes ±20% (689 × 12). There were 804/8,268 harmful/potentially harmful changes, 922/8,268 beneficial/potentially beneficial changes and 6,542/8,268 opportunities for change where none occurred. Therefore, looking at harmful/potentially harmful changes there were 804/8,268 (9.7%, 95% CI for SEM 9.1 to 10.4). Looking at potentially beneficial changes this occurred in 922/8,268 (11.2%, 95% CI for SEM 10.5 to 11.9), and if these were combined with the nonsignificant changes this gave a proportion of 7,464/8,268 (90.3%, 95% CI SEM 89.6 to 90.9). Therefore Hypothesis 2 is rejected.

## Conclusion

The results above are encouraging, especially given the stringent definitions of instability used. By making multiple time-period comparisons the validity of the claims of haemodynamic stability are enforced, compared to previous papers. The number of sessions and measurement points combine to add weight to our findings, supported by robust confidence interval data.

